# Development of a novel immunoassay to detect interactions with the transactivation domain of p53: application to screening of new drugs

**DOI:** 10.1038/s41598-017-09574-7

**Published:** 2017-08-23

**Authors:** Yufeng Xiong, Yingsong Wu, Shuhong Luo, Yang Gao, Yujing Xiong, Daxiang Chen, Hao Deng, Wenbo Hao, Tiancai Liu, Ming Li

**Affiliations:** 1 State Key Laboratory of Organ Failure, Institute of Antibody Engineering, School of Laboratory Medicine and Biotechnology, Southern Medical University, Guangzhou, 510515 China; 2 Guangdong Provincial Key Laboratory of Tropical Disease Research, School of Laboratory Medicine and Biotechnology, Southern Medical University, Guangzhou, 510515 China; 3 Department of Laboratory Medicine, School of Stomatology and Medicine, Foshan University, 5 Hebin Road, Chancheng District, Foshan, Guangdong Province 528000 P. R. China; 4 Department of Obstetrics and Gynaecology, Prince of Wales Hospital, Chinese University of Hong Kong, Shatin, 999077 Hong Kong

## Abstract

Tumor protein p53 acts as a trans-activator that negatively regulates cell division by controlling a set of genes required for cell cycle regulation, making it a tumor suppressor in different types of tumors. Because the transcriptional activity of p53 plays an important role in the occurrence and development of tumors, reactivation of p53 transcriptional activity has been sought as a novel cancer therapeutic strategy. There is great interest in developing high-throughput assays to identify inhibitors of molecules that bind the transcription-activation domain of p53, especially for wt p53-containing tumors. In the present study, taking MDM2 as an example, a novel amplified luminescent proximity homogeneous assay (AlphaLISA) was modified from a binding competition assay to detect the interactions between the transcription-activation domain of p53 and its ligands. This assay can be adapted as a high-throughput assay for screening new inhibitors. A panel of well-known p53-MDM2 binding inhibitors was used to validate this method, and demonstrated its utility, sensitivity and robustness. In summary, we have developed a novel protein-protein interaction detection immunoassay that can be used in a high-throughput format to screen new drug candidates for reactivation of p53. This assay has been successfully validated through a series of p53-MDM2 binding inhibitors.

## Introduction

The p53 protein, the guardian of the genome, plays an essential role in the regulation of cell cycle, apoptosis and DNA repair by defending cells against various cellular stresses, such as hypoxia and DNA damage^1–3^. P53 with impaired function can no longer protect the integrity of cell the genome and these cells are able to pass mutations to the next generation. As such, it is not surprising that p53 is associated with human tumor occurrence and growth^4^. Globally, there are approximately 22 million patients suffering from different kinds of cancer that are affected by p53^5^. Approximately half of these patients bear wild-type p53 in tumor cells, but its function is impaired by negative regulators through degradation or inhibition^6^. Among these negative regulation motifs, binding of the transactivation domain (TAD) of p53, thus blocking its transcriptional activity, is crucial^7, 8^.

The full TAD of p53 is found in residues 1-93 and is composed of three subdomains including TAD1 (residues 1–40), TAD2 (residues 41–61), and the proline-rich domain (residues 61–93)^9–11^. Certain proteins have been found to bind one or both of the TAD domains and thereby inhibit p53 transcriptional activity. For example, it is well known that MDM2 is representative of a p53-negative regulator in which the N-terminal domain directly binds the TAD1 of p53 via a putative helix formed by residues 18–26^12^. Thus, reactivation of p53 by displacing MDM2, or other negative regulators, from wt p53 in cancer cells remains a goal for drug discovery in oncology. To date, some compounds, including nutlins^13^, spirooxindoles^14^ and benzodiazepinediones^15^, have been reported to disrupt MDM2 binding to the TAD of p53, but few studies target other p53-negative regulators, such as MDMX. In terms of tumor treatment, inhibitors targeting MDM2 or other negative regulators could be highly effective^16, 17^. Accordingly, it is necessary to identify cellular proteins that interact with the TAD of p53 and develop corresponding inhibitors to reactivate p53, which is an attractive therapeutic strategy for cancer therapy.

The purpose of this study is to develop a homogenous immunoassay, termed an AlphaLISA, for specifically monitoring total free p53 TAD, which can be widely used to detect the TAD binding to a variety of regulators via competition assay. Furthermore, this detection method could be applied to screen new inhibitors that disrupt the binding and reactivate p53. Because there is no need for blocking and washing steps, this homogenous assay is time- and labor-saving, and amenable to miniaturization in 384-well plate format for high-throughput screening^18, 19^. In contrast to the traditional approaches requiring purified proteins, AlphaLISA is not affected by other proteins in the cell lysate, making it much more convenient than traditional assays^20–23^. Here, we used MDM2 as an example to develop an AlphaLISA assay to measure interactions with p53 and further validated its ability to screen potential inhibitors by successfully identifying known p53-MDM2 binding inhibitors, such as Nutlin-3a.

## Results and Discussion

### Characterization of p53 TAD domain binding to the MDM2 ligand

**Figure 1:**
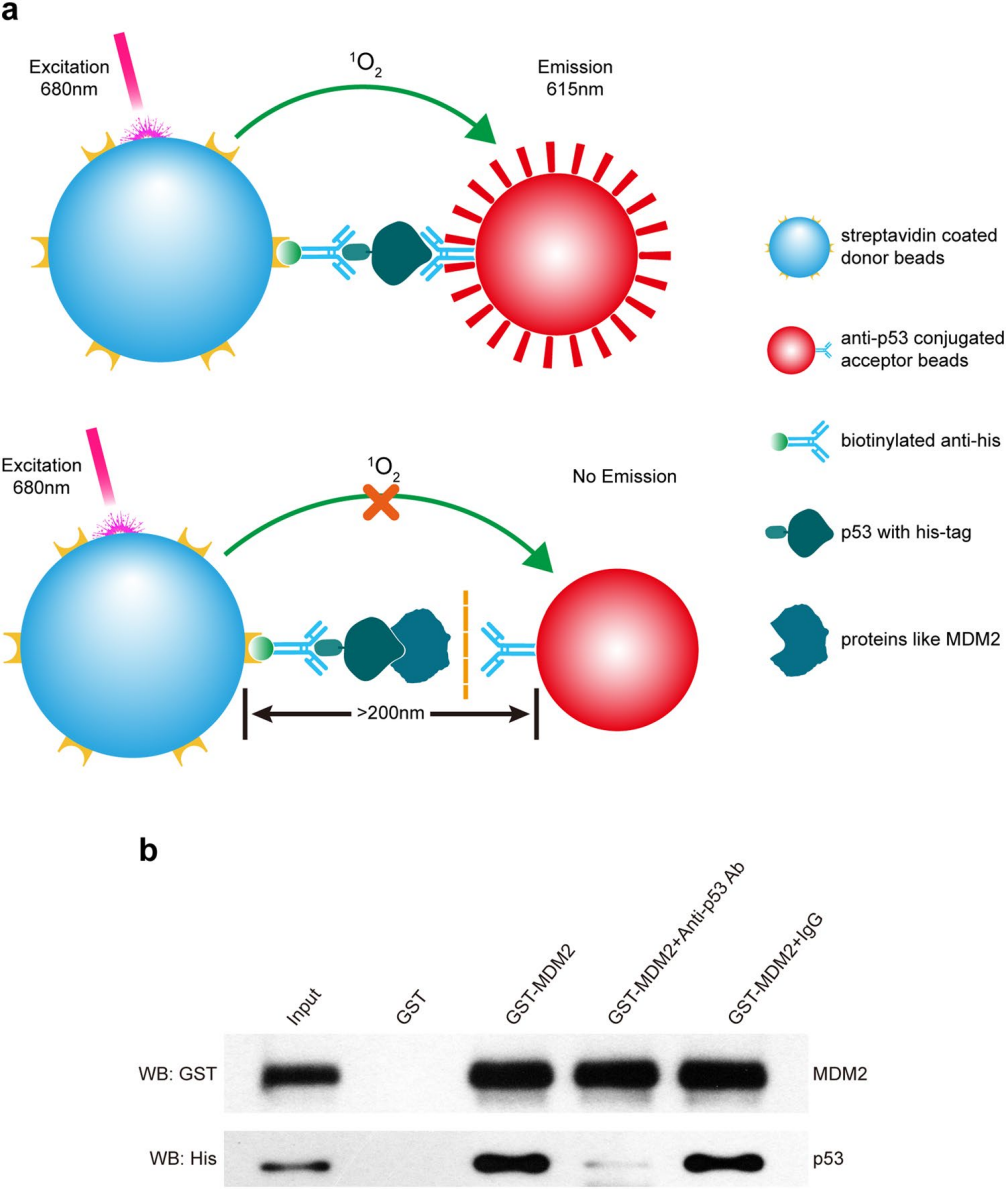
Analysis of the interaction between p53-His and MDM2-GST in cellular extracts. (**a**) A schematic diagram for detection of recombinant protein p53-His in cells using AlphaLISA. Acceptor beads that are directly conjugated to anti-p53 antibodies capture the TAD of p53. Streptavidin coated donor beads capture the immune complex between the biotinylated anti-His antibodies and His tag, bringing donor beads into close proximity to the acceptor beads. After excitation at 680 nm, acceptor beads receive singlet oxygen from donor beads and produce fluorescent emission at 615 nm. As shown in the lower image, if ligands like MDM2, which can block the TAD domain of p53, exist, acceptor beads are not able to capture p53, resulting in no emission. (**b**) GST pull-down assays were performed with pENTER-p53 transfected HEK293T cell lysates as prey and MDM2-GST as bait. Immunoblotting was performed using anti-His and anti-GST antibodies. p53-His was pulled down by MDM2-GST but not by GST. When anti-p53 antibodies were added to the cell lysates, p53-His pulled down by MDM2-GST was remarkably reduced. However, isotype IgG had no effect.

The aim of our work was to establish a universal AlphaLISA assay to detect the interactions between the p53 TAD and its ligands, such as MDM2 and MDM4. In the AlphaLISA assay, donor beads and acceptor beads were connected, with the help of anti-His and anti-p53 antibodies, to p53-His protein, limiting the distance between donor bead and acceptor bead to less than 200 nm. Upon illumination at 680 nm, singlet oxygen produced by the donor beads diffused into the conjunct acceptor beads, resulting in the emission of light at 615 nm (Fig. 1a upper panel). However, if proteins like MDM2, which compete with anti-p53 antibody to interact with p53 TAD domain, are present in the solution, as shown in Fig. 1a lower panel, there are no acceptor beads in close proximity to the donor beads, and therefore no signal. Thus, the competition between anti-p53 antibody and proteins like MDM2 is a key challenge when developing this AlphaLISA assay.

Prior to the development of AlphaLISA assay, we performed experiments to validate the binding of p53 to MDM2 in the cellular extracts. Full-length p53 protein tagged with His at the C-terminus was isolated from HEK293T cells transfected with pEnter-p53, while MDM2 tagged with GST at the N-terminus was expressed in a prokaryotic system. As shown by western blot assays (Fig. 1b), p53-His could be pulled down by MDM2-GST but not by GST. This result indicates that p53 physically interacts with MDM2 in cellular extracts, which is consistent with previous studies^24–26^.

To characterize further the ability of the anti-p53 DO-1 antibody to disrupt the interaction between p53 and MDM2, we utilized another GST pulldown experiment. The reason for choosing anti-p53 DO-1 antibody was that it could recognize a part of the TAD at the N-terminal epitope mapping between amino acid residues 11-25 of human p53^27^. Indeed, the addition of anti-p53 DO-1 antibody led to a significant decrease in the amount of p53-His pulled down by MDM2-GST, whereas isotype IgG had no effect (Fig. 1b). Altogether, GST pulldown experiments suggest that p53-His, MDM2-GST and anti-p53 DO-1 antibody are a suitable system for development of TAD binding assays to support screening of new drugs.

### Set-up of AlphaLISA to detect p53-His in cellular extracts

**Figure 2:**
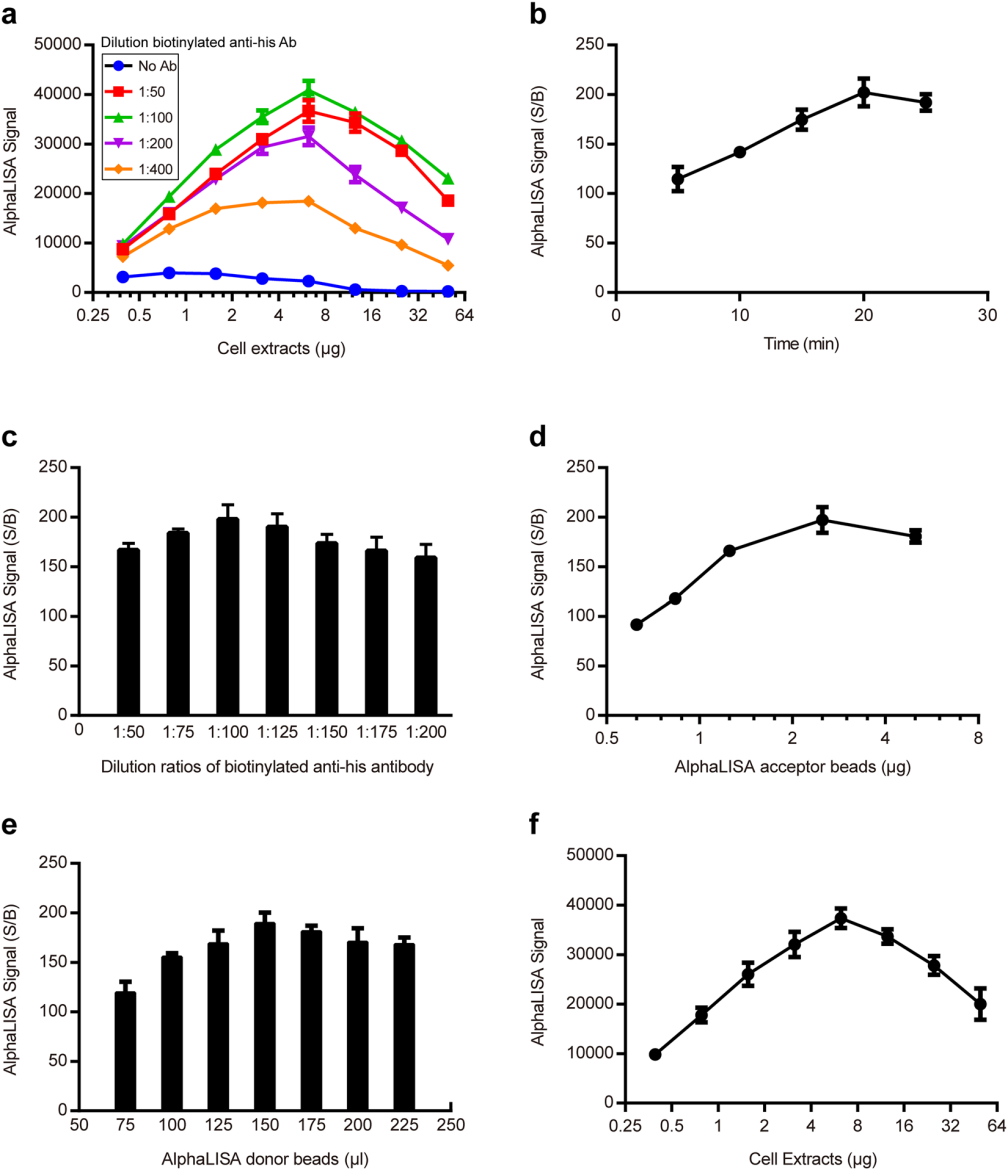
Development of AlphaLISA assay to detect recombinant protein p53-His in cellular extracts. (**a**) A matrix cross-titration of a panel of cell-extract amounts against a dilution series of biotinylated anti-His antibodies revealed the optimal cell-extract amount for subsequent experiments. (**b**) The incubation time of acceptor beads, the biotinylated anti-His antibodies and cell extracts was tested between 5 and 25 min. AlphaLISA signal (S/B) is represented as the ratio of AlphaLISA signal in the presence and absence of cell extracts. For subsequent experiments an incubation time of 20 min (S/B = 202) was adopted. (**c**) Titration of biotinylated anti-His antibody with the optimal amount of cell extracts and incubation time. (**d**,**e**) Titration of AlphaLISA acceptor and donor beads at previously determined optimal conditions. (**f**) Dose-response curve of cell extracts containing p53-His. Each point is an average of three independent experiments.

In order to set up the competition AlphaLISA, a conventional sandwich immunoassay to detect p53-His must first be developed. In a pretest, we evaluated the optimal amount of HEK293 cellular extracts containing p53-His using a matrix cross-titration of biotinylated anti-His antibody and HEK293 cellular extracts containing p53-His. Acceptor beads (2.5 μg/well) were incubated with differing amounts of biotinylated anti-His antibodies and HEK293 cellular extracts for 15 minutes^28^, after which donor beads (175 μl/well) were added. After a 15 minutes incubation, the AlphaLISA signals were detected and plotted against the concentration of biotinylated anti-His antibodies and HEK293 cellular extracts. As expected, the hook-effect, which was common when the beads were saturated with p53-His, was observed when the concentration exceeded 6.25 μg/well for HEK293 cellular extracts containing p53-His (Fig. 2a). In order to maintain a high assay signal and sensitivity, we selected 6.25 μg/well as the optimal HEK293 cellular extract concentration for subsequent experiments.

Because the immunoreaction time significantly influenced the development of fluorescence intensity, we studied the incubation time of acceptor beads, biotinylated anti-His antibodies and HEK293 cellular extracts. Experiments were carried out by adding 6.25 μg/well HEK293 cellular extracts with p53-His to acceptor beads and biotinylated anti-His antibodies. To create negative controls, the same amount of HEK293 cellular extracts, without p53-His, from HEK293 cells transfected with pEnter was used. AlphaLISA signals were measured every 5 min over the period of 5–25 min from the start of incubation, and the signal-to-background ratio (S/B) was calculated as a ratio of the AlphaLISA signal with positive and negative controls. Figure 2b shows that the S/B increases rapidly at first and reaches a maximum at 20 min. Hence, we selected 20 min as the immunoreaction time for further studies.

The amount of biotinylated anti-His antibody, acceptor beads conjugated with anti-p53 antibody and streptavidin-coated donor beads all affected the performance of the AlphaLISA assay. Therefore, a large series of different component concentrations was tested to determine optimal values of these components using the 20-minute reaction time. A range of biotinylated anti-His antibodies diluted in assay buffer (1:50, 1:75, 1:100, 1:125, 1:150, 1:175, 1:200) was explored. As shown in Fig. 2c, the S/B increased as the biotinylated antibodies decreased, but began falling when the dilution ratio was greater than 1:100 because the concentration of biotinylated antibodies was too low to bring p53-His and donor beads together. The dilution ratio of 1:100 for biotinylated anti-His antibody was chosen for use in all subsequent experiments. Various concentrations of acceptor beads ranging from 0.625 to 5 μg/well were tested to determine the optimal concentration. The resulting titration curve, shown in Fig. 2d, showed the hook effect when the concentration exceeded 2.5 μg/well. Therefore, a titration of the donor beads with 2.5 μg/well acceptor beads was performed (Fig. 2e). The S/B reached a maximum at a concentration of 150 μl/well. Consequently, the concentrations chosen for the acceptor and donor beads were 2.5 μg/well and 150 μl/well, respectively.

Additional parameters, such as the assay buffer and cell lysis buffer, were also analyzed and described in the Methods section. Ultimately, dose-response experiments using cellular extracts containing p53-His were performed using the optimized conditions. As shown in Fig. 2f, the dose-response curve revealed that the AlphaLISA assay developed was sensitive enough to detect differing amounts of p53-His in cellular extracts. Because the AlphaLISA assay is homogeneous, p53-His could be rapidly detected far more conveniently compared with conventional immunoassays, such as ELISA and western blot^29–31^. Additionally, the use of anti-p53 DO1 antibody allowed us to monitor total free TAD of p53-His, specifically, in cellular extracts. To our knowledge, this is the only homogeneous detection assay suitable for the high-throughput screening of ligands of the p53 TAD.

### Development of competitive binding AlphaLISA to detect the interaction between p53 TAD and its ligands

**Figure 3:**
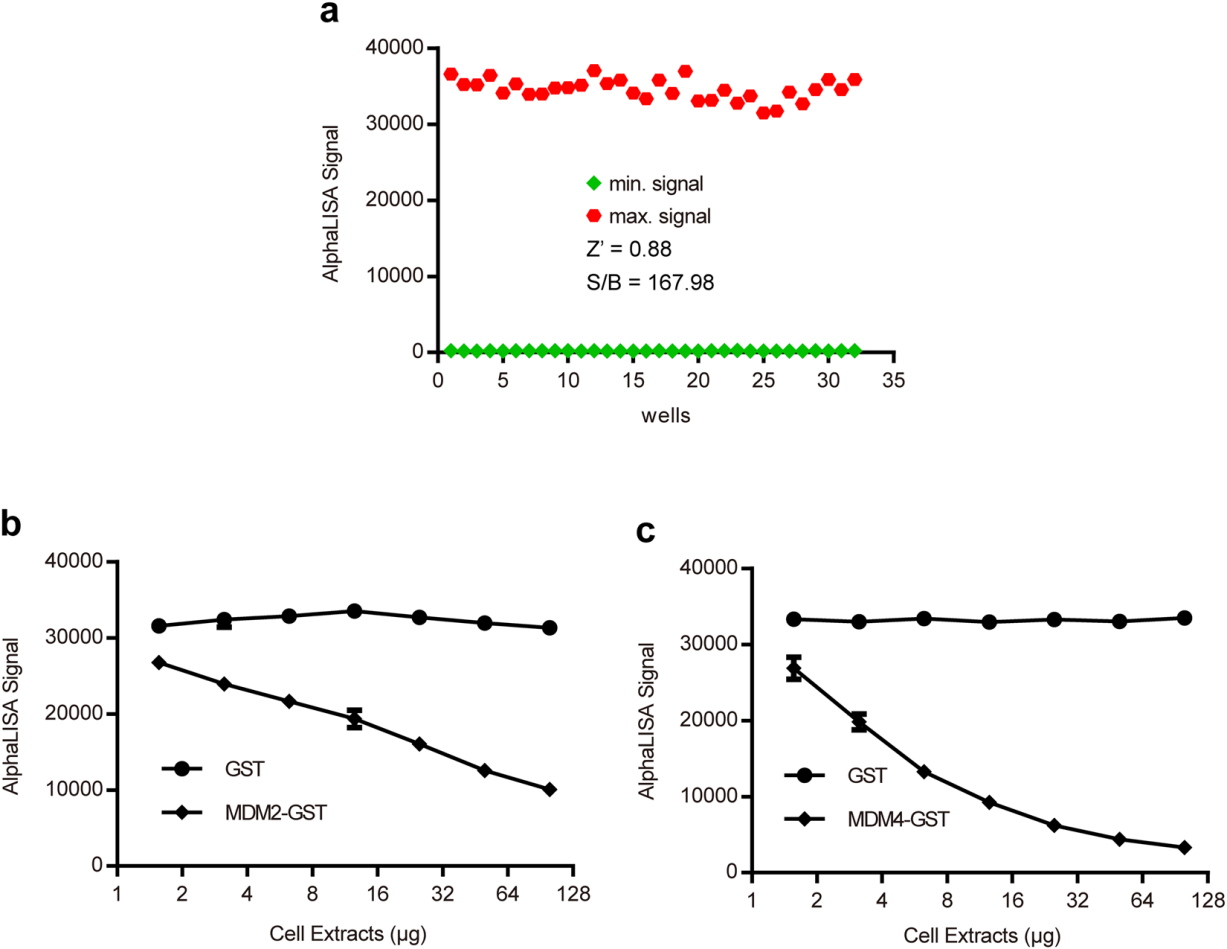
AlphaLISA assay development to detect the interaction between p53 TAD and its ligands in HTS format and validation of the assay. (**a**) Robustness of the AlphaLISA assay. In order to determine Z′-factor, an experiment including 32 positive controls with optimal amount of HEK293T cell extracts (min. signal) and 32 negative controls with same amount of cell extracts containing p53-His (max. signal) was conducted on 96-well plates. (**b**,**c**) Dose-response curve of cell extracts containing MDM2-GST/MDM4-GST, which compete with anti-p53 antibody for binding to the TAD of p53.

The goal of the present study was to use AlphaLISA for the screening of new ligands of the p53 TAD. We measured the displacement of TAD from the anti-p53 antibody/p53-His complex by inhibiting this interaction with ligands of the TAD, such as MDM2, which should ultimately result in a decrease of the AlphaLISA signal. In order to assess the robustness of the ligand screening assay, Z′ factor (index of assay quality) and signal-to-background (S/B) ratio were calculated using a control plate into which 32 maximum controls using HEK293 cellular extract containing p53-His and 32 minimum controls using HEK293 cellular extract without p53-His were dispensed (Fig. 3a). The Z′ factor and signal-to-background ratio were calculated to be 0.88 and 167.98, respectively, which demonstrated that this AlphaLISA assay was robust and suitable for HTS^32^.

A competitive binding assay was used to validate the interaction between the p53 TAD and MDM2. A serial dilution series of cellular extracts containing MDM2-GST in assay buffer was prepared. The AlphaLISA assay was conducted in which the mixture, including acceptor beads, HEK293 cellular extracts containing p53-His, biotinylated anti-His antibody and cellular extracts containing MDM2-GST, was incubated at 37 °C for 20 min. Donor beads were added and the mixture was incubated for another 15 min. As a negative control, cellular extracts containing MDM2-GST were substituted by cellular extracts containing GST. The AlphaLISA signals were plotted against cellular extracts as shown in Fig. 3b. The dose response curve showed that the AlphaLISA signals decreased as the cellular extracts containing MDM2-GST increased, while the AlphaLISA signals of the negative control remained unchanged. Cellular extracts containing MDM4-GST were utilized to validate the interaction between p53 TAD and MDM4^33–35^, in the same manner as described for MDM2. As shown in Fig. 3c, the AlphaLISA signals decreased rapidly over the range from 1.56 to 6.25 μg, but slowed when the amount exceeded 6.25 μg. In short, these dose response curves indicated a significant competition of MDM2/MDM4 with anti-p53 antibody for binding the TAD of p53.

Compared with conventional assays utilized to detect protein-protein interaction, such as co-immunoprecipitation and GST pulldown, the AlphaLISA assay presented in this study was sensitive, fast (35 min), and simple to perform. Compared with Tandem affinity purification (TAP) and Surface Plasmon Resonance (SPR), this assay was user-friendly, inexpensive, and had acceptable sensitivity. The main advantage of this assay, compared with other methods, is that this technology is particularly well suited for high-throughput applications in cellular extracts and does not require purification.

### Pharmacological validation of inhibitor screening AlphaLISA using known inhibitors of the p53-MDM2 interaction

**Figure 4:**
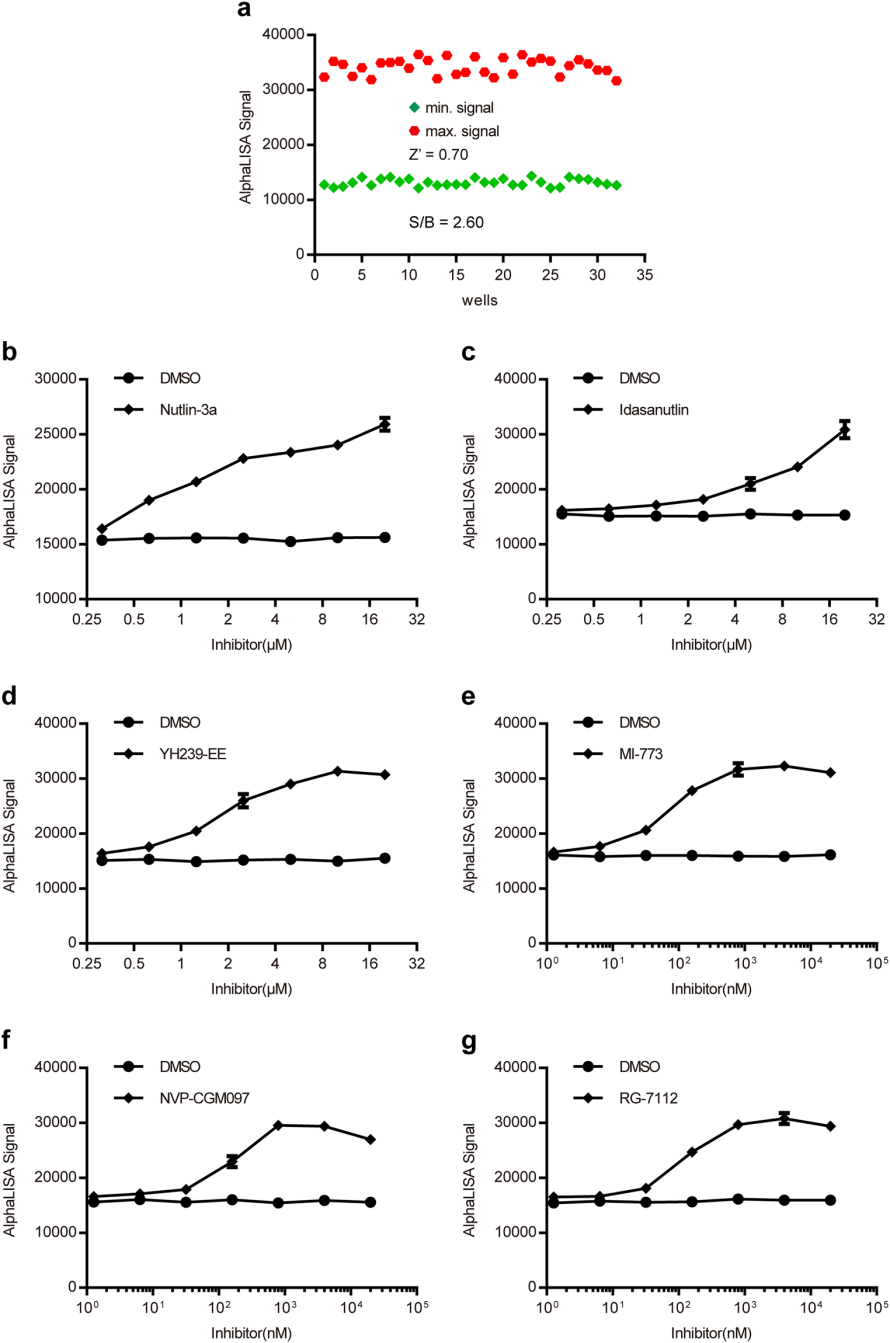
Development of an AlphaLISA based MDM2-inhibitor screening assay and pharmacological validation of the assay. (**a**) Robustness of the MDM2-inhibitor screening assay. AlphaLISA signals of 32 positive controls with optimal amount of cell extracts containing GST (max. signal) and 32 negative controls with the same amount of cell extracts containing MDM2-GST (min. signal) were plotted. (**b**–**g**) Dose-response curves for a few MDM2 inhibitors.

HTS assays developed for drug discovery should be validated for pharmacological relevance and for assay performance. In the AlphaLISA assay utilized to screen inhibitors of the p53-MDM2 interaction, six different inhibitors (Table 1) known to disrupt p53-MDM2 binding^13, 36–40^ were tested at different concentrations to verify that they could release p53 TAD from MDM2, leading to increase in AlphaLISA signals. This would also validate that the assay was sensitive enough to detect the changes in a robust manner. As with the competitive binding assay, Z′ factor and signal-to-background ratio were calculated using 32 minimum controls using cellular extracts containing MDM2-GST and 32 maximum controls using cellular extracts containing GST. To reduce the amount of MDM2-GST used, while maintaining appropriate signal and sensitivity, 33.3 μg/well cellular extracts was used for the AlphaLISA assay. As shown in Fig. 4a, the Z′ factor and signal-to-background ratio were 0.70 and 2.60, respectively, indicating that the assay was robust.

**Table 1:** Inhibitors used in the inhibitor screening assay.

Inhibitor	Structure	MW/Formula/CAS No.	Description
Nutlin-3a		581.49 C_30_H_30_Cl_2_N_4_O_4_ 4675576-98-4	The active enantiomer of Nutlin-3, inhibits the p53/MDM2 interaction with IC50 of 90 nM in a cell-free assay.
Idasanutlin		616.48 C_31_H_29_Cl_2_F_2_N_3_O_4_ 1229705-06-9	Idasanutlin (RG-7388) is a potent and selective p53-MDM2 inhibitor with IC50 of 6 nM.
YH239-EE		504.41 C_25_H_27_Cl_2_N_3_O_4_ 1364488-67-4	YH239-EE, the ethyl ester of YH239, is a potent p53-MDM2 antagonist and an apoptosis inducer.
MI-773		562.50 C_29_H_34_Cl_2_FN_3_O_3_ 1303607-60-4	MI-773 (SAR405838) is an orally available MDM2 antagonist with Ki of 0.88 nM. Phase 1.
NVP-CGM097		659.26 C_38_H_47_ClN_4_O_4_ 1313363-54-0	NVP-CGM097 is a highly potent and selective MDM2 inhibitor, disrupting the interaction between both proteins.
RG-7112		726.28 C_38_H_48_Cl_2_N_4_O_4_S 939981-39-2	RG7112 (RO5045337) is an orally bioavailable and selective p53-MDM2 inhibitor. Phase 1.

To validate the capacity of this assay to screen inhibitors, six inhibitors of the p53-MDM2 interaction were prepared at a final concentration of 20 mM in DMSO and stored at −80 °C. On the basis of preliminary experiments, serial dilutions of nutlin-3a, idasanutlin and YH239-EE at a ratio of 1:5 starting with a 20 μM concentration were prepared in assay buffer, and MI-773, NVP-CGM097 and RG-7112 were prepared at a ratio of 1:10. Serial dilutions of inhibitors were added into the mixture of acceptor beads, cellular extracts containing p53-His, biotinylated antibody and cellular extracts containing MDM2-GST to generate a dose response curve. Serial dilutions of DMSO were added to gain the basal level of p53-MDM2 without inhibitor. All of the dose response curves depicted in Fig. 4 displayed increasing AlphaLISA signals along with increasing dosage of different inhibitors, but no change was seen using the DMSO negative control. Notably, while the signals of MI-773, NVP-CGM097 and RG-7112 ultimately reached a plateau when the concentration exceeded 1 μΜ, the response signals of nutlin-3a, idasanutlin and YH239-EE continued to rise rapidly. These results strongly suggest that the six inhibitors known to bind MDM2 can release the p53 TAD and that this HTS inhibitor screening assay had acceptable sensitivity to detect the changes of AlphaLISA signal caused by the actions of the inhibitors.

### Confirming specificity of inhibitor screening AlphaLISA

**Figure 5:**
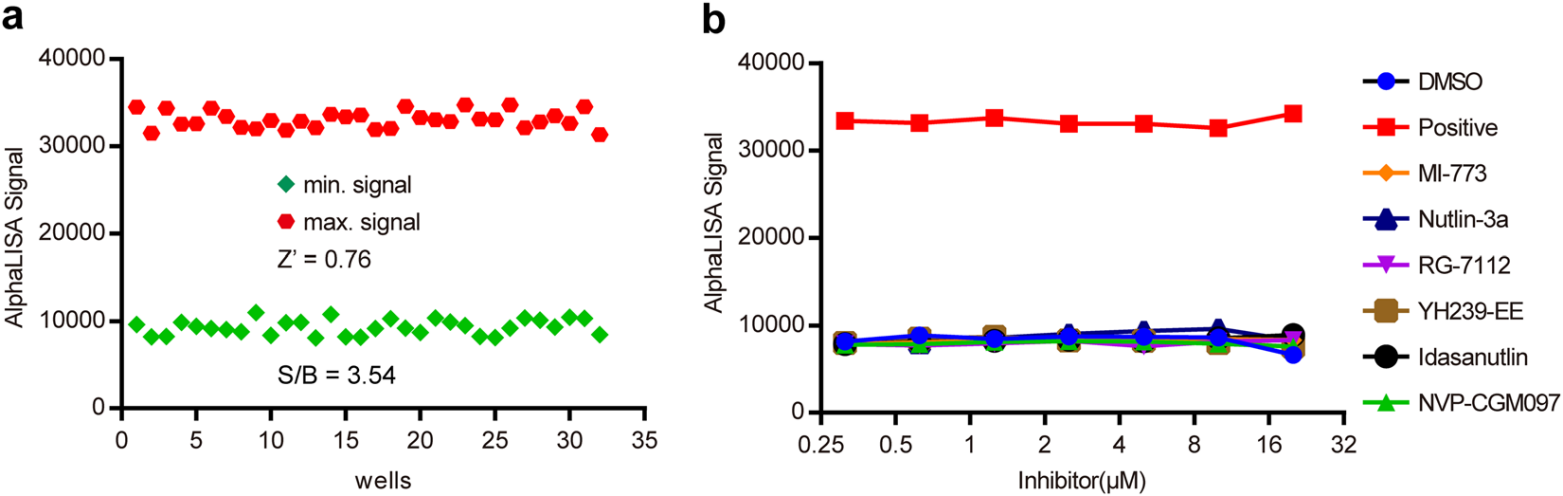
Development of an AlphaLISA based MDM4-inhibitor screening assay and the specificity of the assay. (**a**) Robustness of the MDM4-inhibitor screening assay. AlphaLISA signals of 32 positive controls with optimal amounts of cell extracts containing GST (max. signal) and 32 negative controls with the same amount of cell extracts containing MDM4-GST (min. signal) were plotted. (**b**) Dose-response curves for a few MDM2 inhibitors.

To evaluate the specificity of the inhibitor screening assay, the six inhibitors of MDM2 used previously were examined to evaluate inhibition of p53-MDM4 interaction. The Z′ factor and signal-to-background ratio were 0.76 and 3.54, respectively, calculated from 32 minimum controls and 32 maximum controls in which 12.5 μg/well cellular extracts containing GST/MDM4-GST were used to achieve appropriate signal and sensitivity. These data indicated the suitability of this AlphaLISA assay for the HTS evaluation of inhibitors of p53-MDM4 interaction (Fig. 5a). The results of the assay conducted to test the six MDM2 inhibitors, shown in Fig. 5b, show that the AlphaLISA signals remain unchanged as the dose of inhibitor rose, identically to the signals of the DMSO negative control, indicating that inhibitors of MDM2 have no ability to affect the signal and that this AlphaLISA assay is highly specific.

## Conclusion

The tumor suppressor protein p53 is a transcription factor that can be negatively regulated by its ligands, such as MDM2, through binding to the transcription domain of p53 in tumors^41^. Blocking interactions with MDM2 is a prime strategy for new cancer drug development. Multiple methods have been utilized to support the discovery of inhibitors of MDM2^42^, but the development of assays for protein-protein interactions involving TAD domains has historically been slow, as has the development of inhibitor screening assays for other negative regulators.

In the present study, we have developed a novel, high-throughput screening amenable AlphaLISA assay to discover molecules in cellular extracts that interact with the transactivation domain of p53. This assay is simple, rapid, and more applicable to high-throughput formats, compared with conventional methods to identify protein-protein interactions. Importantly, the assay time is significantly reduced as compared to a sandwich AlphaLISA assay format^43^. Furthermore, this AlphaLISA assay was implemented as a universal method to screen rapidly inhibitors of targets that specifically bind to the p53 TAD. Although this inhibitor screening assay, based on cellular extracts, exhibited acceptable QC parameters, further improvement is needed to increase assay sensitivity and to develop this platform further as a cell-based assay. Altogether, we believe that the AlphaLISA immunoassay demonstrated in this work may have wide applications for the further development of anticancer therapeutics.

## Methods

### Reagents and Plasmids

4-morpholineethanesulfonic acid (MES), Sodium cyanoborohydride (NaBH_3_CN), Tween-20, Carboxy-methoxy lamine (CMO), Proclin-300, Dimethylsulfoxide (DMSO), Biotinyl-N-hydroxy-succinimide (NHS-Biotin), protease inhibitor cocktail and PMSF were purchased from Sigma-Aldrich (St. Louis, MO, USA). Streptavidin-coated donor beads and unconjugated europium-acceptor beads were purchased from Beyondbiotech (Shanghai, China). Anti-p53 DO-1 and anti-MDM2 N-20 antibodies were obtained from Santa Cruz Biotechnology (Santa Cruz, CA, USA), and anti-His monoclonal antibody was acquired from Bioworld Technology (St. Louis, USA). The open reading frame (ORF) encoding p53 was generated by RT-PCR amplification from 293T cells and subcloned into a pEnter plasmid (ViGene Biosciences, Rockville, USA) carrying a C-terminal 6 × his tag. GST (glutathione transferase) recombinant pGEX-4T-MDM2 plasmid was a kind gift from Mien-Chie Hung (The University of Texas, Austin, TX, USA)^44^. The sequences of both recombinant plasmids generated were verified by sequencing prior to use. All other reagents used were of analytical reagent grade and a Milli-Q water purification system (Millipore, MA, USA) was employed to supply ultra-pure water used throughout the experiments.

### Cell culture and transfection

Human embryonic kidney cells (293T) were cultured at 37 °C (5% CO_2_) in DMEM supplemented with 10% fetal bovine serum (FBS) and 1% GlutaMAX. 24 h prior to transfection, 1 × 10^6^ cells were seeded in a 10 cm^2^ dish and transfected when about 70% confluent. After incubation for 10 minutes at room temperature, the dilution containing 10 µg pEnter-p53 and 20 µL jetPRIME transfection agent in 500 µL jetPRIME buffer was added to the cells and the dish was returned to the incubator. Four hours later, the transfection medium was replaced with fresh culture medium. Cells were cultured for 48 h post-transfection, washed twice with cold PBS and lysed with 1 ml of cell lysis buffer (20 mM Tris (pH 7.5), 150 mM NaCl, 1% Triton X-100) containing PMSF and a protease inhibitor cocktail. After centrifugation at 20,000 × g for 20 min at 4 °C, the supernatant was aliquoted and stored at −80 °C until use.

### Preparation of GST-MDM2 protein

To express recombinant MDM2 tagged with GST, Escherichia coli BL21 (DE3) cells carrying pGEX-4T-MDM2 were grown in LB medium at 37 °C for 4 h. When the OD600 was about 0.8, the cells were induced with 1 mM IPTG overnight at 18 °C. Bacteria were collected by centrifugation at 5000 × g (15 min, 4 °C) and washed three times under the same conditions. The pellets were resuspended in PBS and lysed by three freeze-thaw cycles followed by sonication with 100 µM PMSF. Finally, the cellular extract was obtained by centrifugation at 20,000 × g for 20 min at 4 °C to remove cellular debris. After identification by Coomassie blue staining, the supernatant was divided into Eppendorf tubes and stored at −80 °C.

### Pulldown assay

To determine the interaction between p53 and MDM2 in vitro, a GST pulldown assay was preformed following the protocol described previously^45^. Briefly, Glutathione Sepharose 4B beads bound with GST or GST-MDM2 fusion protein were incubated with HEK-293T cellular extracts containing p53 with 6 × his tag for 3 h at 4 °C. After being washed three times, the bound proteins were eluted by boiling for 10 min and p53 protein content was analyzed by western blot. In order to identify competition of the anti-p53 DO-1 antibodies with MDM2 in binding to p53, another GST pulldown experiment was conducted as described above. The only difference between two experiments is that 2 µg anti-p53 DO-1 antibodies were added to the HEK-293T cellular extracts containing p53-His protein.

### Biotinylation of anti-His antibodies

A 10 mM solution of NHS-Biotin was prepared by dissolving 3.4 mg NHS-Biotin in 1 ml DMSO. 100 µg anti-His monoclonal antibodies diluted in 196.7 µl PBS (pH 7.4) were mixed with 3.3 µl of the NHS-Biotin solution at a 1:50 molar ratio (antibody: biotin) and incubated for 2 h at room temperature. In order to remove excess biotin, the biotinylated antibodies were washed three times with PBS using Amicon Ultra centrifugal filters. The final concentration was adjusted to 0.5 mg/ml with PBS containing 0.1% NaN_3_.

### Conjugation of anti-p53 antibodies to AlphaLISA acceptor beads

In order to conjugate anti-p53 antibodies to acceptor beads, 10% Tween-20, a fresh 400 mM NaBH_3_CN solution and 1 mg/ml antibodies were prepared in 100 mM MES buffer (pH 6.0). After washing the acceptor beads twice, 1 mg beads, 1.25 µl 10% Tween-20, 10 µl NaBH_3_CN solution and anti-p53 antibodies were mixed together and the total reaction volume was adjusted to 200 µl with MES buffer. The reaction solution was briefly sonicated and incubated at 37 °C using a rotary shaker. After 48 h, 10 µl fresh 65 mg/mL solution of carboxymethoxylamine (CMO) in 800 mM NaOH was added to the reaction and incubated for 1 hour to block the unreacted sites. The mixture was centrifuged at 15,000 g for 10 min and the bead pellet was washed thrice with PBS buffer. Finally, the acceptor beads were resuspended in 200 µl PBS buffer with 0.05% Proclin-300.

### AlphaLISA detection assay

All AlphaLISA experiments were performed in white 96-well plates with an optimized assay buffer containing 25 mM Hepes (pH 7.4), 100 mM NaCl, 0.5% BSA, 0.1% Tween-20, 2 mg/ml Dextran-500, and 0.01% Proclin-300. Two sequential incubations were required in the assay which was initiated with the mixture of 25 µl each of conjugated acceptor beads, biotinylated anti-His antibodies and samples per well. The samples used in different experiments were prepared as follows: (a) different amounts of HEK293 cellular extracts containing p53-His used in the p53 detection assay; (b) 6.25 μg HEK293 cellular extracts containing p53-His and different amounts of cellular extracts containing GST-MDM2 used in the binding competition assay; (c) 6.25 μg HEK293 cellular extracts containing p53-His, 33.3 μg cellular extracts containing GST-MDM2 and different concentrations of inhibitors used in the inhibitor screening assay. After the first incubation of the mixture at 37 °C for 20 min, streptavidin-coated donor beads were added into the reaction solution and incubated under subdued lighting conditions at 37 °C for another 15 min. The AlphaLISA signals were measured with the Light Initiated Chemiluminescent Assay (LiCA) system (Beyond Diagnostics) with excitation and emission wavelengths set at 680 nm and 615 nm, respectively. The data of three independent trials in each experiment were analyzed and the results were plotted using GraphPad Prism. In order to optimize the assay, the signal-to-background ratio (S/B) was used to normalize multiple experiments. The Z′-factor calculated using the equation Z′ = 1 − (3 × SD of positive control + 3 × SD of negative control)/|mean of positive control–mean of negative control| was used to estimate the assay quality.

### Data Availability

The datasets generated during and/or analysed during the current study are available from the corresponding author on reasonable request.
